# PD-L1 blockade improves survival in experimental sepsis by inhibiting lymphocyte apoptosis and reversing monocyte dysfunction

**DOI:** 10.1186/cc9354

**Published:** 2010-11-30

**Authors:** Yan Zhang, Ying Zhou, Jingsheng Lou, Jinbao Li, Lulong Bo, Keming Zhu, Xiaojian Wan, Xiaoming Deng, Zailong Cai

**Affiliations:** 1 Clinical Research Center, Changhai Hospital, Second Military Medical University, 168 Changhai Road, Shanghai, 200433, PR China; 2 Department of Biochemistry and Molecular Biology, Second Military Medical University, 800 Xiangyin Road, Shanghai, 200433, PR China; 3 Department of Anesthesiology, Changhai Hospital, Second Military Medical University, 168 Changhai Road, Shanghai, 200433, PR China

## Abstract

**Introduction:**

Lymphocyte apoptosis and monocyte dysfunction play a pivotal role in sepsis-induced immunosuppression. Programmed death-1 (PD1) and its ligand programmed death ligand-1 (PD-L1) exert inhibitory function by regulating the balance among T cell activation, tolerance, and immunopathology. PD-1 deficiency or blockade has been shown to improve survival in murine sepsis. However, PD-L1 and PD-1 differ in their expression patterns and the role of PD-L1 in sepsis-induced immunosuppression is still unknown.

**Methods:**

Sepsis was induced in adult C57BL/6 male mice via cecal ligation and puncture (CLP). The expression of PD-1 and PD-L1 expression on peripheral T cells, B cells and monocytes were measured 24 hours after CLP or sham surgery. Additionally, the effects of anti-PD-L1 antibody on lymphocyte number, apoptosis of spleen and thymus, activities of caspase-8 and caspase-9, cytokine production, bacterial clearance, and survival were determined.

**Results:**

Expression of PD-1 on T cells, B cells and monocytes and PD-L1 on B cells and monocytes were up-regulated in septic animals compared to sham-operated controls. PD-L1 blockade significantly improved survival of CLP mice. Anti-PD-L1 antibody administration prevented sepsis-induced depletion of lymphocytes, increased tumor necrosis factor (TNF)-α and interleukin (IL)-6 production, decreased IL-10 production, and enhanced bacterial clearance.

**Conclusions:**

PD-L1 blockade exerts a protective effect on sepsis at least partly by inhibiting lymphocyte apoptosis and reversing monocyte dysfunction. Anti-PD-L1 antibody administration may be a promising therapeutic strategy for sepsis-induced immunosuppression.

## Introduction

Sepsis, a systemic inflammatory response to infection, results in the death of more than 210,000 people in the United States annually [1]; it remains the leading cause of death in critical ill patients [2]. Because critical care treatment is becoming expensive, understanding the molecular mechanisms underlying the development of sepsis is important in identifying new therapeutic strategies.

Protracted immunosuppression caused by impaired pathogen clearance after primary infection or susceptibility to secondary infection may contribute to the high rates of morbidity and mortality associated with sepsis [3,4]. Accumulating evidence [5-7] suggests the pivotal role of apoptosis in sepsis-induced immunosuppression. Numerous studies have shown that the numbers of peripheral and splenic lymphocytes are reduced during sepsis in both humans and animals [8,9]. Apoptosis is known to be mainly responsible for decreased lymphocyte numbers, and the extent of lymphocyte apoptosis correlates with the severity of sepsis [10]. In multiple animal models of sepsis, survival rates have been remarkably improved by inhibiting lymphocyte apoptosis by using selective caspase inhibitors [11,12]; altering proapoptotic/antiapoptotic protein expression [13,14]; treatment with survival promoting cytokines such as interleukin (IL)-7 [15] and/or IL-15 [16]; and modulating costimulatory receptors [17,18].

Monocytes play an essential role in innate immune defense against microbial infection. rapidly exhibit an impaired production of proinflammatory cytokines in response to additional bacterial challenge [19], and a reduced antigen presentation capacity likely due to their decreased expression of human leukocyte antigen(locus)DR (HLA-DR) [20]. Such monocytic deactivation indicates a state of globally impaired immune functions and correlates with poor clinical outcome in critically ill patients.

Programmed death-1 (PD-1) is a newly defined co-inhibitory receptor whose expression can be induced, primarily on the cell surface of activated CD4 and CD8 T cells. PD-1 has two main ligands: PD-L1 (B7-H1) and PD-L2 (B7-DC). PD-L1 is broadly expressed on hematopoietic and non-hematopoietic cells, including T cells, B cells, dendritic cells (DCs), macrophages, endothelial cells, epithelial cells, pancreatic islet cells, and fibroblastic reticular cells [21]. PD-1 and its ligand exert inhibitory effects in the setting of persistent antigenic stimulation by regulating the balance among T cell activation, tolerance, and immunopathology. The PD-1/PD-L1 pathway plays a critical role in the regulation of autoimmunity, tumor immunity, transplantation immunity, allergy, immune privilege, and ischemia/reperfusion injury [22]. Recent findings suggest that the PD-1/PD-L1 pathway plays an important role in the interaction between host and pathogenic microbes that evolved to resist immune responses. Those pathogens include viruses [23], certain bacteria [24], fungi [25], and some worms [26]. Studies using PD-L1-knockout mice support the finding that PD-L1 is the primary regulatory counter receptor for the inhibitory function of PD-1 [27]. Many studies showed that PD-L1 antagonism can block the interaction of PD-1 and PD-L1 [28-31]. Hence, we hypothesized that the blockade of PD-L1 using anti-PD-L1 antibody would improve survival in sepsis. The purpose of this study was to elucidate the effect of PD-L1 blockade caused by an antagonistic antibody to PD-L1 on survival in a murine cecal ligation and puncture (CLP) model of sepsis. In addition, this study attempted to determine the potential mechanism underlying the putative beneficial effect of PD-L1 antagonism in sepsis.

## Materials and methods

### CLP model of sepsis

All experiments were approved by the Institutional Animal Care and Use Committee. Adult 8- to 10-week-old (22 to 30 g) C57BL/6 male mice were purchased from the Animals Experimentation Center of Second Military Medical University. CLP-induced polymicrobial sepsis was performed as described previously [15]. Briefly, mice were anesthetized with isofluorane and a midline abdominal incision was made. The cecum was mobilized, ligated below the ileocecal valve, and punctured twice with a 22 gauge needle to induce polymicrobial peritonitis. The abdominal wall was closed in two layers. Sham-operated mice underwent the same procedure, including opening the peritoneum and exposing the bowel, but without ligation and needle perforation of the cecum. After surgery, the mice were injected with 1 mL physiologic saline solution for fluid resuscitation. All mice had unlimited access to food and water both pre- and postoperatively.

### PD-1 and PD-L1 expression on peripheral T cells, B cells and monocytes

Mice were euthanized 24 h after CLP or sham-operated surgery, and blood was obtained to analyze expression of PD-1 and PD-L1. After erythrocytes were lysed with lysing solution (BD Bioscience San Jose, CA, USA), cells were stained with fluorochrome-conjugated anti-CD3, anti-CD19, anti-CD11b, anti-PD-1 or anti-PD-L1 antibodies. Flow cytometric analysis was performed on a MACS Quant (Miltenyi Biotech, Bergisch Gladbach, Germany) using Flowjo software version 7.6 (Tree Star, Ashland, OR, USA). For flow cytometric analysis, we first gated on a lymphocyte/monocyte population in FSC/SSC, then T cells, B cells or monocytes were gated on CD3, CD19 or CD11b-positive cells, respectively. Abs were purchased from eBioscience (San Jose, CA, USA): CD3-PerCP-Cy5.5 (Clone: 145-2C11), CD19-PE-Cy7 (Clone: 1D3), CD11b-APC (Clone: M1/70), PD-1-PE (Clone: J43), PD-L1-PE (Clone: MIH5).

### Effect of PD-L1 blockade on the survival of septic mice

In order to compare the effect of anti-PD-L1 antibody administration at different time-points on survival, treatment with the antibody before or after CLP was used. To confirm the *in vivo *protective effect of PD-L1 blockade on sepsis, C57BL/6 male mice were intraperitoneally injected with anti-PD-L1 antibody (50 μg/mouse), isotype antibody (50 μg/mouse), or saline 24 h before CLP, and survival rates were assessed over the subsequent eight days. To assess the potential therapeutic effect of PD-L1 blockade, mice that underwent CLP were subsequently randomized to receive intraperitoneal anti-PD-L1 antibody (50 μg/mouse), isotype control antibody (50 μg/mouse) or saline 3 h after CLP surgery. Survival was over the subsequent eight days. All mice were subcutaneously administered 1 mL normal saline within 30 minutes after CLP and allowed free access to food and water.

### Determination of lymphocytes counts in blood, spleen and thymus

Mice that underwent CLP were randomized to intraperitoneally receive anti-PD-L1 antibody (50 μg/mouse), isotype control antibody (50 μg/mouse), or saline after CLP surgery. The blood, spleen and thymus of the septic and sham-operated mice were harvested 24 h after CLP. The total cell number was counted after lysis of erythrocytes (for spleen and thymus, single-cell suspension was prepared). Cells were also stained with fluorochrome-conjugated antibodies to cell subset-specific surface markers (CD3 for T cells and CD19 for B cells). Lymphocyte numbers were calculated by obtaining total cell count and lymphocyte subgroup percentage by FACS analysis.

### Quantification of apoptosis in the spleen and thymus

The spleen and thymus were harvested from septic and sham-operated mice 24 h after surgery and treatment, and fixed with 10% buffered formalin. Terminal deoxynucleotidyl transferase-mediated dUTP nick end labeling (TUNEL) staining was performed using the ApopTag Plus Peroxidase In Situ Apoptosis Detection Kit (Chemicon Billerica, MA, USA) according to the manufacturer's instructions. In brief, sections were incubated in equilibration buffer for 10 minutes and then terminal deoxynucleotidyl transferase and dUTP-digoxigenin were added to the sections and incubated in a 37°C humidified chamber for 1 h. The reaction was then stopped and the slices were washed and incubated with anti-digoxigenin-peroxidase solution, colorized with DAB/H_2_O_2_, and counterstained with bis-benzamide. From each specimen, two sections were initially examined under light microscopy at low magnification (×100). Five fields per section were randomly examined at a higher magnification (×400). Two investigators examined the samples microscopically in a blinded fashion. The percentage of the TUNEL-positive cells was used to determine the apoptosis rate. For detection of T cells apoptosis and activities of caspase-8 and caspase-9 in the thymus, thymuses from CLP or sham-operated mice were harvested 24 h after surgery and treatment. A single-cell suspension was prepared. CD3^+ ^T cells were stained with annexin V and propidium iodide (PI) for apoptosis measurement and stained for active caspase-8 using IEHD-FMK (BioVision, Mountain View, CA, USA), and for active caspase-9 using LEHD-FMK (BioVision, USA) according to the manufacturer's instructions.

### Cytokine analysis and bacterial clearance

Plasma from CLP or sham-operated mice was harvested 24 h after surgery and treatment. Concentrations of tumor necrosis factor (TNF)-α, IL-6, and IL-10 were measured using murine enzyme-linked immunosorbent assay (ELISA) kit (R&D Systems, Minneapolis, MN, USA) according to the manufacturer's instructions. For bacterial clearance, blood and peritoneal lavage fluid samples were collected 24 h after surgery and treatment. Blood was collected by heart puncture after isoflurane anesthesia. Peritoneal lavage fluid was harvested after injecting 2 mL PBS into the peritoneum and serial dilution in samples was serially diluted to 10-, 100-, or 1,000-fold in 500 μL PBS. A 100 μL aliquot of each dilution was spread on a tryptic soy agar (TSA) blood agar plate. All plates were incubated at 37°C for 24 h. Colonies were counted and expressed as colony forming units (CFUs)/mL for all the samples.

### Statistical analysis

Data reported are the mean (SEM). All statistical analyses were performed using Prism 4.0 (GraphPad Software, La Jolla, CA, USA). Survival of the two subgroups was estimated by Kaplan-Meier analysis; comparisons were performed by the log-rank test. All comparisons among groups were performed by Mann-Whitney analysis of variance. For multigroup analysis, intergroup comparisons were performed by Dunn's test. Significance was accepted at *P *< 0.05.

## Results

### Upregulation of PD-1 and PD-L1 on T cells, B cells and monocytes during sepsis

**Figure 1:**
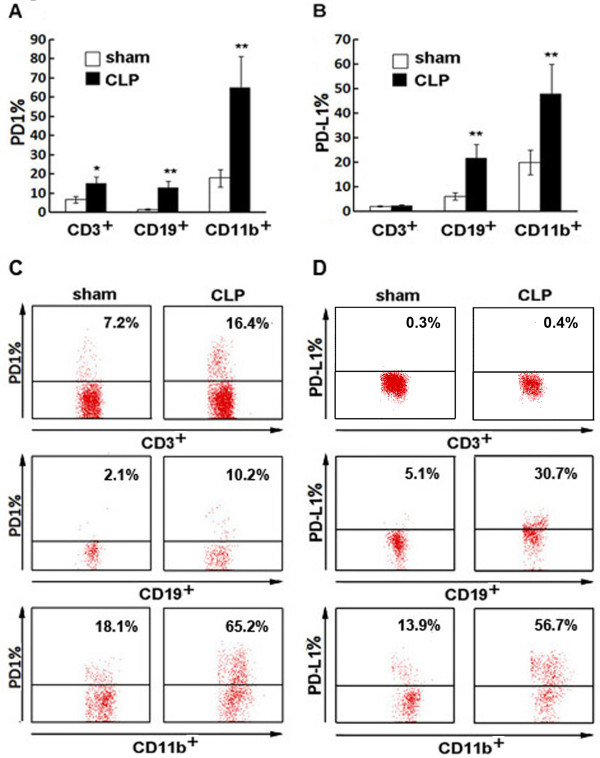
**PD-1 and PD-L1 expression on T cells, B cells and monocytes**. **(A) **Percent of PD-1 expression on CD3^+ ^T cells, CD19^+ ^B cells and CD11b^+ ^monocytes 24 h after CLP (*n *= 5) or sham control surgery (*n *= 5). **(B) **Percent of PD-L1 expression on CD3^+ ^T cells, CD19^+^B cells and CD11b^+ ^monocytes 24 h after CLP (*n *= 5) or sham control surgery (*n *= 5), **(C, D) **Representative PD-1 and PD-L1 expression on T, B cells and monocytes detected by flow cytometry. * *P <*0.05, ** *P <*0.01.

Expression of PD-1 on T cells (CD3^+^), B cells (CD19^+^) and monocytes (CD11b^+^) increased significantly in CLP mice compared with sham mice (*P *< 0.05 and *P *< 0.01, respectively) (Figure 1A, C). Expression of PD-L1 on B cells and monocytes were also upregulated in septic animals (*P *< 0.01), whereas PD-L1 expression on T cells was not altered significantly (Figure 1B, D).

### PD-L1 blockade improves survival of murine sepsis

**Figure 2:**
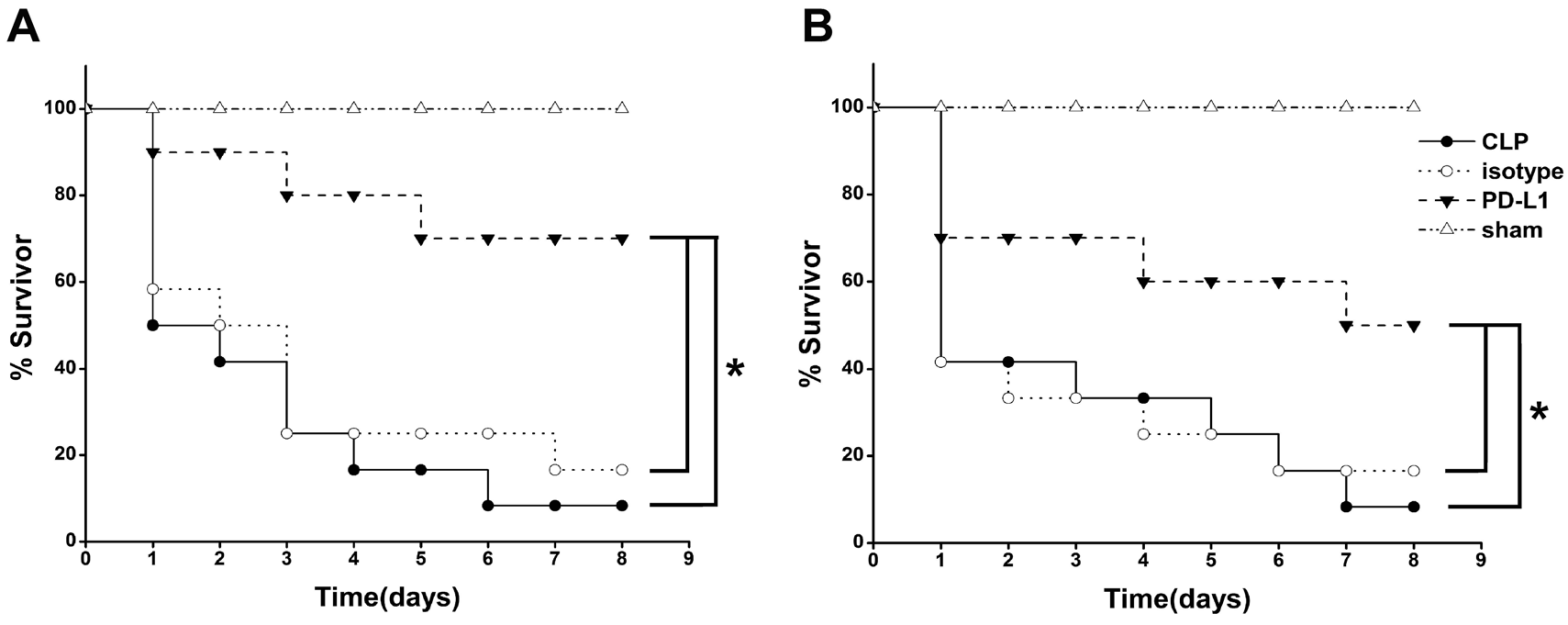
**Anti-PD-L1 antibody administration protects mice from sepsis-induced lethality**. **(A) **Anti-PD-L1 antibody pretreatment protected mice from CLP. CLP mice were given 50 μg anti-PD-L1 antibody (*n *= 18), 50 μg isotype control antibody (*n *= 12) or 0.2 mL saline intraperitoneally 24 h before CLP surgery. **(B) **Effect of intraperitoneal anti-PD-L1 antibody treatment given 3 h after CLP. CLP mice were given 50 μg anti-PD-L1 antibody (*n *= 18), 50 μg isotype control antibody (*n *= 12) or 0.2 mL saline (*n *= 12) intraperitoneally 3 h after CLP. Survival was monitored for eight days. Data are shown as the survival percent of animals. *P <0.05.

Mice pretreated with anti-PD-L1 antibody for 24 h before CLP showed an improved eight-day survival (70.0%) compared with that of mice pretreated with saline (7.8%; *P *< 0.05) or those pretreated with isotype control antibody (16.6%; *P *< 0.05; Figure 2A). Mice treated with anti-PD-L1 antibody 3 h after CLP also showed an improved eight-day survival (50.0%, *P *< 0.05; Figure 2B). No statistical difference was shown between mice treated with isotype controls or with saline.

### PD-L1 blockade decreases lymphocyte apoptosis in spleen and thymus of septic mice *in situ*

**Figure 3:**
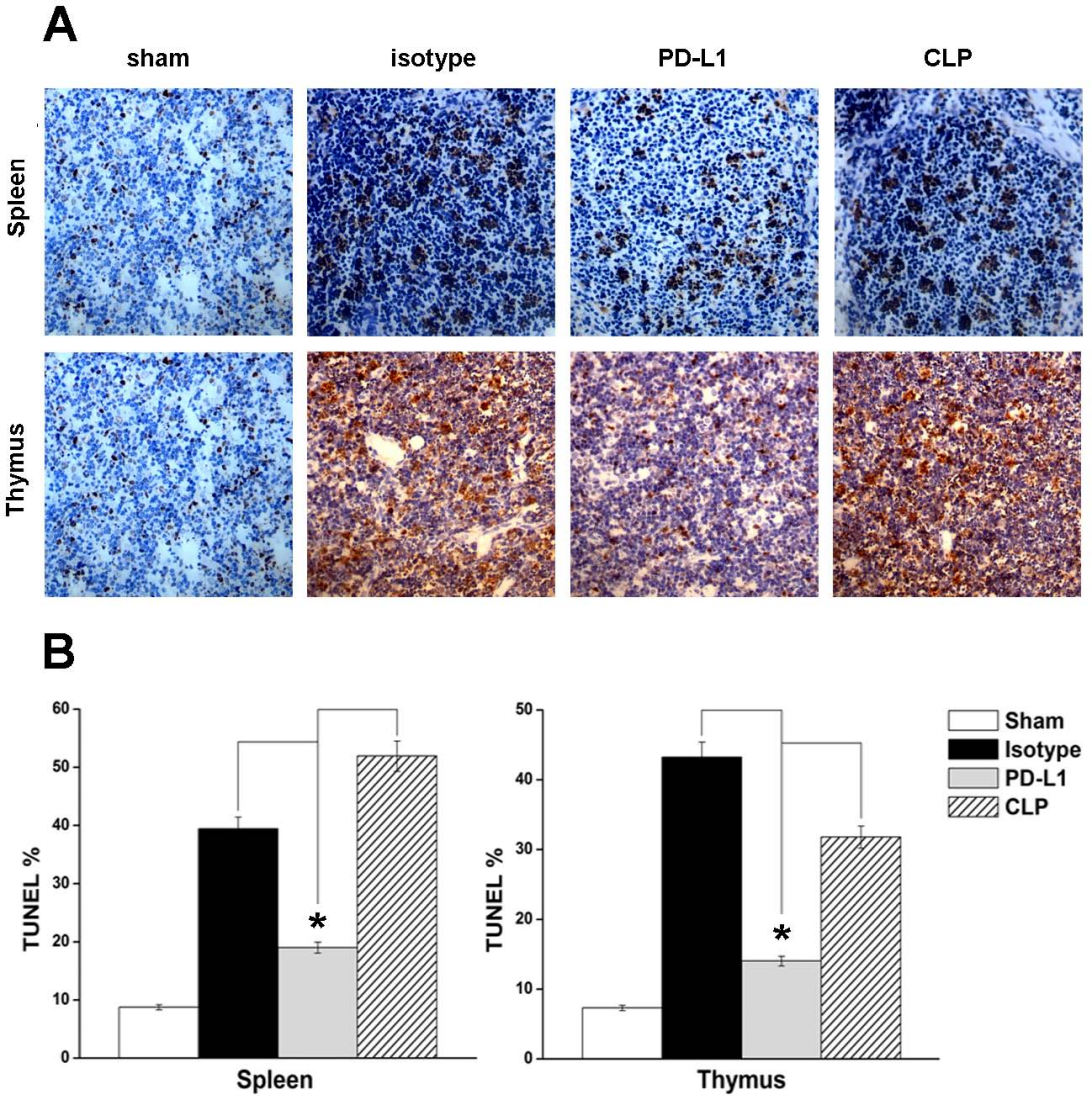
**PD-L1 blockade inhibits cell apoptosis in spleen and thymus**. Mice underwent a sham procedure, CLP, CLP plus anti-PD-L1 or isotype administration (*n *= 5 for each group). Spleen and thymus were harvested 24 h after surgery. **(A) **Representative sections analyzed by an *in situ *TUNEL assay. **(B) **Percent of the TUNEL-positive cells is used to show the cell apoptosis in spleen and thymus of the 4 groups. * *P <*0.05.

In the spleen of sham-operated mice, physiologic TUNEL-positive cells, which were morphologically identical to lymphocytes, were sporadically found, and their number increased markedly after induction of sepsis by CLP. However, the CLP mice that were administered anti-PD-L1 antibody showed decreased numbers of TUNEL-positive cells. There was a reduction in the number of apoptotic lymphocytes in the thymus of mice treated with anti-PD-L1 antibody compared with those in the mice treated with CLP-only or in those treated with the isotype antibody (Figure 3A, B).

### PD-L1 blockade increases lymphocyte number in peripheral blood, spleen and thymus

**Figure 4:**
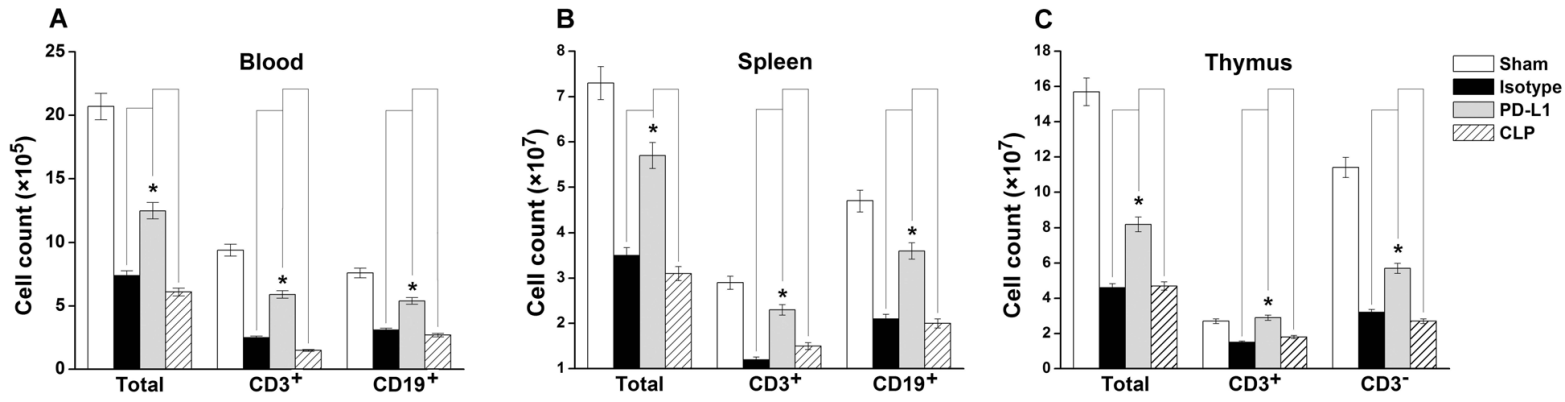
**Cell numbers in blood (A), spleen (B) and thymus (C)**. Mice underwent a sham procedure, CLP, CLP plus anti-PD-L1 administration, or CLP plus isotype administration (*n *= 5 for each group). Blood, thymus and spleen were harvested 24 h after surgery. The total cell number was counted after lysis of erythrocytes (for spleen and thymus, preparation of a single-cell suspension was required). Lymphocyte numbers (CD3+ T cells, CD3- T cells, CD19+ B cells) were calculated by the total number and percent of lymphocyte subgroups resulting from FACS analysis, respectively. * *P <*0.05.

As expected, the numbers of total white blood cells and lymphocytes were higher in the blood of the PD-L1 blockade group than those in the CLP-only or isotype antibody control group. Similar results were noted in the spleens and thymuses (Figure 4).

### Both the extrinsic death receptor pathway and the intrinsic mitochondrial-mediated pathway contribute to decreased lymphocyte apoptosis *in vivo*

**Figure 5:**
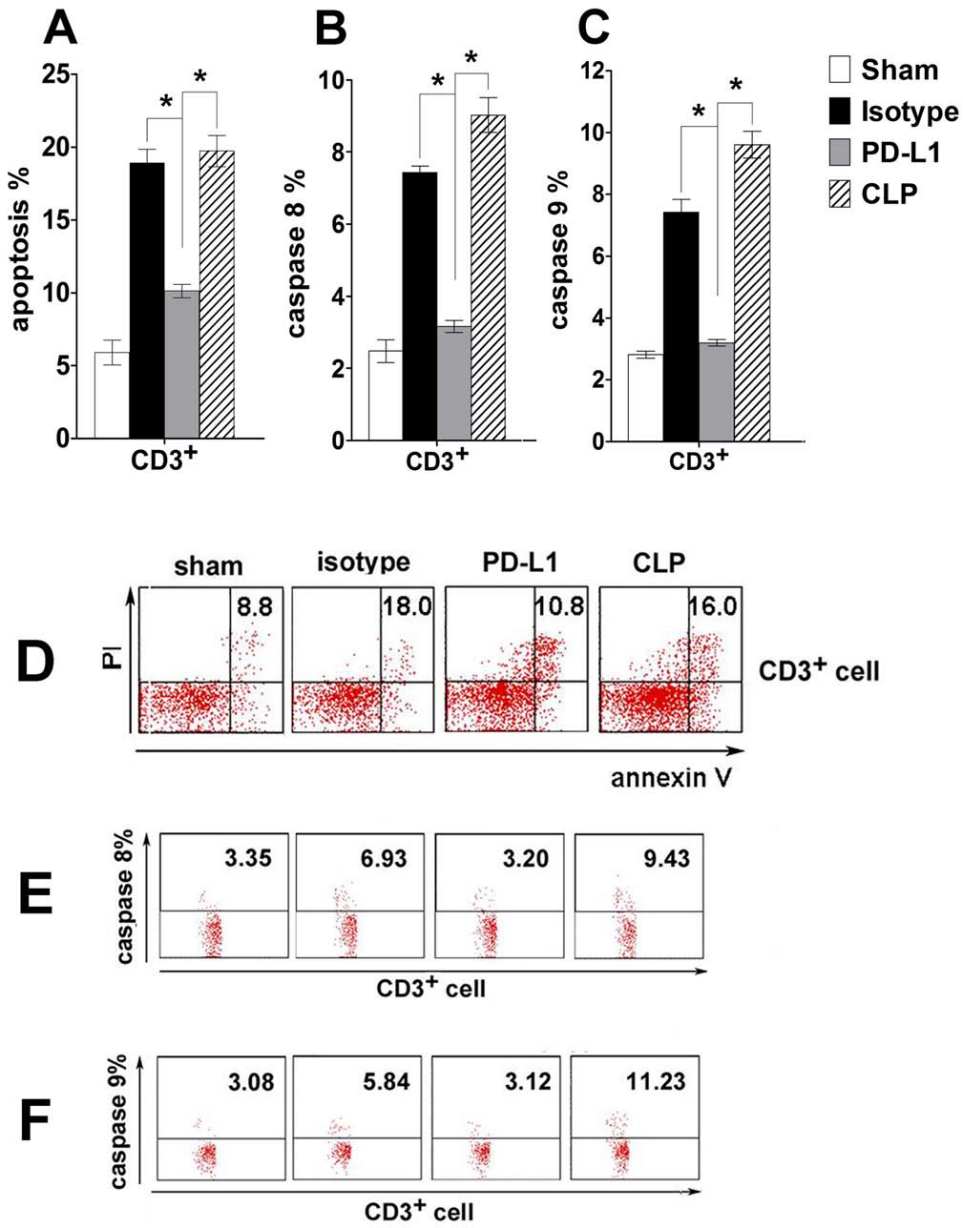
**Both extrinsic and intrinsic pathways contribute to decreased lymphocyte apoptosis *in vivo***. Mice underwent sham procedure, CLP, CLP plus anti-PD-L1 antibody administration, or CLP plus isotype administration. Thymus was harvested 24 h after surgery and stained for annexin V and propidium iodide (PI) **(A, D) **or FITC-labeled IEHD-FMK **(B, E) **or LEHD-FMK **(C, F) **which can irreversibly binds to activated caspase-8 or activated caspase-9. **(D)**, **(E)**, and **(F) **are the representative flow cytometry dot plots. Values in the upper right quadrant indicate the percent of apoptotic cells, caspase-8 or caspase-9 positive cells, respectively. * *P <*0.05.

Data showed that PD-L1 blockade decreased the apoptosis of CD3^+ ^T cells in the thymus (Figure 5A, D). PD-L1 blockade also decreased the activity of caspase-8 to approximately 30% of CLP level (Figure 5B, E). Similar results were also observed for active caspase-9 (Figure 5C, F). These data indicate that both the extrinsic death receptor pathway and the intrinsic pathway contributed to the decreased lymphocyte apoptosis *in vivo*. This is consistent with the widely accepted idea that the extrinsic and the intrinsic apoptotic pathways are intimately connected.

### Cytokines levels and bacterial clearance

**Figure 6:**
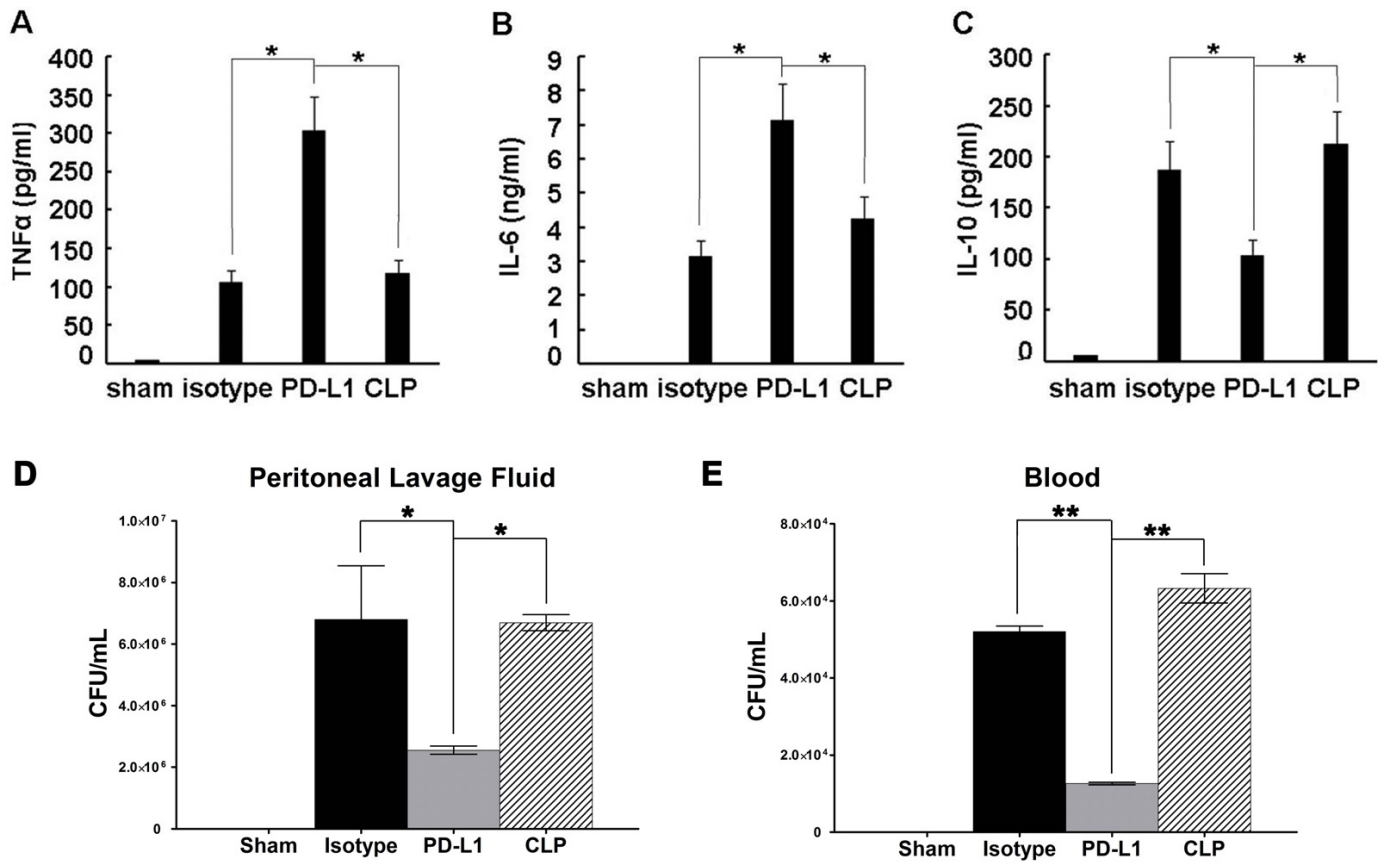
**Levels of plasma cytokines and bacterial clearance**. Mice underwent a sham procedure, CLP, CLP plus anti-PD-L1 administration, or CLP plus isotype control administration (*n *= 5 for each group). Levels of TNF-α **(A)**, IL-6 **(B) **and IL-10 **(C) **were measured 24 h after surgery. Treatment with anti-PDL1 antibody improves bacterial clearance in septic mice. Mice that received anti-PD-L1 antibody exhibited a deceased bacterial burden in peritoneal lavage fluid in comparison with mice that received isotype antibody or saline **(D)**, Mice that received anti-PD-L1 antibody exhibited a deceased bacterial burden in blood in comparison with mice that received isotype antibody or saline **(E)**, * *P *< 0.05, ** *P *< 0.01.

PD-L1 blockade significantly increased the expression of TNF-α, IL-6 and decreased the level of IL-10 in CLP murine plasma (Figure 6A, B, C). Mice that received anti-PD-L1 antibody showed a deceased bacterial burden in both blood and peritoneal lavage fluid compared with that in the mice treated with the isotype antibody or those treated with saline (Figure 6D, E).

## Discussion

Lymphocytes and monocytes are thought to be critical in mediating both apoptosis and cytokine release during sepsis and the PD-1/PD-L1 pathway is critical in T cell co-stimulatory signal regulation [21,22]. To explore the role of PD-L1 in sepsis, we initially investigated PD-L1 and PD-1 expression on T cells, B cells, and monocytes in response to sepsis. We found a significant up-regulation of PD-L1 expression on monocytes and B cell at 24 h post-CLP. Our finding of increased PD-1 expression is consistent with that of the study by Huang *et al*. [32]. Our study indicated that besides PD-1, PD-L1 expression was also up-regulated on monocyte during sepsis, suggesting that PD-L1 may play a role in the process.

PD-1 deficiency or anti-PD-1 antibody has been shown to improve survival in murine sepsis models [32,33]. Our findings suggest that besides PD-1 blockade, anti-PD-L1 antibody administration significantly improved survival of CLP mice, and decreased T cell apoptosis and improved monocyte dysfunction, which may contribute to the beneficial effect of PD-L1 blockade.

Several studies have shown that PD-L1 blockade augmented T cell functions in chronic virus infection [27,34]. However, our study did not suggest that PD-L1 blockade had significant effects on CD4^+ ^and CD8^+ ^T cell functions, including proliferation and interferon-γ or IL-2 production (data not shown). Our data show that PD-L1 blockade decreased lymphocyte apoptosis in the spleens and thymuses of septic mice *in situ*, increased lymphocyte number in peripheral blood, spleens and thymuses, indicating that like PD-1 blockade, anti-PD-L1 antibody administration could indeed inhibit T cell apoptosis. We further show that both the extrinsic death receptor pathway and the intrinsic mitochondrial-mediated pathway contributed to decreased lymphocyte apoptosis *in vivo*. Of note, we found that PD-L1 blockade also decreased apoptosis of bronchial epithelial cells and alveolar epithelial cell in lungs (data not shown), suggesting perhaps other cell targets involved in the beneficial effect of PD-L1 blockade.

However, inhibition of apoptosis by PD-L1 blockade is incomplete, and T cell apoptosis remains to some degree after PD-L1 pathway blockade, implicating the involvement of other regulatory pathways in sepsis-induced T cell apoptosis.

During the inflammatory response, monocytes present antigens by means of expression of human leukocyte antigen (HLA) receptors and secrete proinflammatory cytokines to amplify the immune response. Multiple studies have demonstrated that during sepsis-induced immunosuppression, monocytes secrete fewer cytokines and down-regulate expression of HLA receptors. This impaired function of monocytes generally predicts increased risk of secondary infection and poor prognosis [35,36]. In our study, we found dramatic up-regulation of PD-L1 on monocytes in CLP mice, and this up-regulation was likely associated with monocyte dysfunction. PD-L1 blockade exhibited a markedly decreased IL-10, elevated TNF-α and IL-6 levels in plasma as well as a decreased bacterial burden both in blood and peritoneal lavage fluid. However, PD-1 blockade did not alter plasma cytokine levels [33].

There is a balance between pro-inflammatory and anti-inflammatory responses during sepsis. Both responses occur simultaneously during the early phase of the disease. During the stage of sepsis-induced immunosuppression, there is an excessive anti-inflammatory response named compensatory anti-inflammatory response syndrome (CARS). CARS has a distinct set of cytokines and cellular responses characterized by the reduction of lymphocytes, decreased cytokine response of monocytes to stimulation, decreased numbers of human leukocyte antigen (HLA) antigen-presenting receptors on monocytes, and expression of anti-inflammatory cytokines such as IL-10 [35]. Our study demonstrated that PD-L1 may play a vital role in the balance of pro-inflammatory and anti-inflammatory responses during sepsis. In addition to a decrease of apoptosis in T cells, PD-L1 blockade could reverse monocyte dysfunction by modulating cytokine production.

## Conclusions

PD-L1 blockade exerts a protective effect on sepsis, at least partly by inhibiting lymphocyte apoptosis and reversing monocyte dysfunction by modulating cytokine production. Anti-PD-L1 antibody administration may be a promising therapeutic strategy for sepsis-induced immunosuppression.

## Key messages

• Expression of PD-1 on T cells, B cells and monocytes and PD-L1 on B cells and monocytes were up-regulated in septic animals.

• PD-L1 blockade significantly improved survival of CLP mice.

• Anti-PD-L1 antibody administration prevented sepsis-induced depletion of lymphocytes, increased TNF-α and IL-6 production, decreased IL-10 production, and enhanced bacterial clearance.

• Anti-PD-L1 antibody administration may be a promising therapeutic strategy for sepsis-induced immuno-suppression.

## Abbreviations

CARS: compensatory anti-inflammatory response syndrome; CFUs: colony forming units; CLP: cecal ligation and puncture; ELISA: enzyme-linked immunosorbent assay; HLA: human leukocyte antigen; IL: interleukin; PD-1: programmed death-1; PD-L1: programmed death ligand-1; PI: propidium iodide; TNF: tumor necrosis factor; TSA: tryptic soy agar; TUNEL: terminal deoxynucleotidyl transferase biotin-dUTP nick end labelling.

## Competing interests

The authors declare that they have no competing interests.

## Authors' contributions

Zhang Y, Zhou Y and LJS contributed equally to this article. They participated in the study design, coordinated the CLP surgery and detected all the samples by flow cytometry, TUNEL and ELISA kits. They also helped to analyze the data and draft the manuscript. LJB helped to design the experiment, analyze the data and draft the manuscript. BLL, WXJ and ZKM helped to analyze the data. CZL and DXM designed the experiment, supervised all of the experimental work and statistical analysis, and wrote the manuscript. All authors read and approved the final manuscript.
